# Processed microalgae: green gold for tissue regeneration and repair

**DOI:** 10.7150/thno.99181

**Published:** 2024-08-19

**Authors:** Sen Liu, Ling Shi, Hailong Luo, Kaiyuan Chen, Meichen Song, Yingjun Wu, Fengzhi Liu, Meng Li, Jie Gao, Yan Wu

**Affiliations:** 1 College of Life Science, Mudanjiang Medical University, Mudanjiang, China.; 2 Department of Neurology, the Affiliated Hongqi Hospital, Mudanjiang Medical University, Aimin District, Mudanjiang 157011, China.; 3 Pathology Department of the Second Affiliated Hospital of Mudanjiang Medical College, Mudanjiang, China.; 4 Department of Dermatology, Shanghai Ninth People's Hospital, Shanghai Jiaotong University, Shanghai, China.; 5 Changhai Clinical Research Unit, Shanghai Changhai Hospital, Naval Medical University, Shanghai, China.; 6 Shanghai Key Laboratory of Nautical Medicine and Translation of Drugs and Medical Devices, Shanghai 200433, China.

**Keywords:** microalgae, processing, tissue damage repair, drug delivery, wound healing

## Abstract

As novel biomedical materials, microalgae have garnered significant interest because of their ability to generate photosynthetic oxygen, their antioxidant activity, and their favorable biocompatibility. Many studies have concentrated on the hypoxia-alleviating effects of microalgae within tumor microenvironments. However, recent findings indicate that microalgae can significantly increase the regeneration of various tissues and organs. To augment microalgae's therapeutic efficacy and mitigate the limitations imposed by immune clearance, it is essential to process microalgae through various processing strategies. This review examines common microalgal species in biomedical applications, such as *Chlorella*, *Chlamydomonas reinhardtii*, diatoms, and *Spirulina*. This review outlines diverse processing methods, including microalgae extracts, microalgae‒nanodrug composite delivery systems, surface modifications, and living microalgae‒loaded hydrogels. It also discusses the latest developments in tissue repair using processed microalgae for skin, gastrointestinal, bone, cardiovascular, lung, nerve, and oral tissues. Furthermore, future directions are presented, and research gaps for processed microalgae are identified. Collectively, these insights may inform the innovation of processed microalgae for various uses and offer guidance for ongoing research in tissue repair.

## Introduction

In the realm of medical science, a pivotal area of investigation is tissue regeneration, which refers to the intricate process by which the body repairs and restores damaged local cells and tissues 1. This process is essential for all living organisms because it maintains tissue integrity and biological function and prevents infections and diseases. The efficacy of tissue regeneration is highly dependent on microenvironmental conditions. As the “soil”, the tissue microenvironment regulates the function of parenchymal cells, which are the “seeds”. Therefore, maintaining the stability of the tissue microenvironment is vital for normal cell proliferation, differentiation, and metabolism. Abnormalities in the extracellular matrix, growth factors, chemokines, or other components in the tissue microenvironment may lead to cell damage 2. Microdamage to local tissues can be alleviated through regulatory mechanisms in the human body. However, impaired tissue repair can lead to delayed or dysregulated wound healing. In such cases, traditional “3Rs” treatment (i.e., resection, repair, and replacement) fails to mimic the regenerative microenvironment. It may result in fibrosis or scarring, which can impair normal tissue function and lead to organ failure and death 2-4. Owing to the drawbacks of traditional therapies, researchers have attempted to develop novel strategies that specifically target the tissue microenvironment to improve tissue regeneration and repair.

The use of microalgae as natural biomedical materials is gradually increasing in the field of tissue regeneration and repair. Microalgae are unicellular or multicellular photosynthetic autotrophic microorganisms widely distributed in seawater and freshwater 5. They consist of lipids, proteins, carbohydrates, and various other components, and their size ranges from a few micrometres to a few hundred micrometres. Therefore, microalgae are small, simple microorganisms with a wide range of sources and low culture costs 6,7. Recent studies have shown that microalgae can regulate microenvironmental conditions at the trauma site during tissue repair and promote wound healing through anti-inflammatory, antioxidative, and antibacterial effects 8-10. In addition, microalgae are rich in various photosynthetic pigments, such as chlorophyll a and b and carotenoids. They can not only efficiently utilize light, carbon dioxide, and water to synthesize oxygen and carbohydrates but also emit fluorescence for imaging purposes. *Chlorophyll*, a natural fluorescent pigment, has an absorption peak at a wavelength of 640-660 nm and can emit red fluorescence when stimulated with a laser. Therefore, microalgae can be used in imaging-guided integrated diagnosis and treatment to monitor the development of lesions continuously and enhance therapeutic efficacy 6,11. Owing to these advantages, microalgae hold great promise as natural biomedical materials in the field of tissue repair. In addition to their applications in the food, nutraceutical, and fuel industries, microalgae are widely used to promote the repair of various tissues, including skin, gastrointestinal, bone, cardiovascular, lung, nerve, and oral tissues. On the basis of in-depth microalgae research, the bioactivity, targeting ability, and functionality of microalgae can be enhanced via the use of microalgae extracts, microalgae-nanodrug composite drug delivery systems, surface modifications, and microalgae-loaded hydrogels. Improving these properties may enhance therapeutic efficacy and promote tissue repair and regeneration. In addition, it can address the limitations imposed on the therapeutic effects of microalgae by these factors, such as microalgae vitality and phagocytosis and clearance by immune cells.

**Figure 1:**
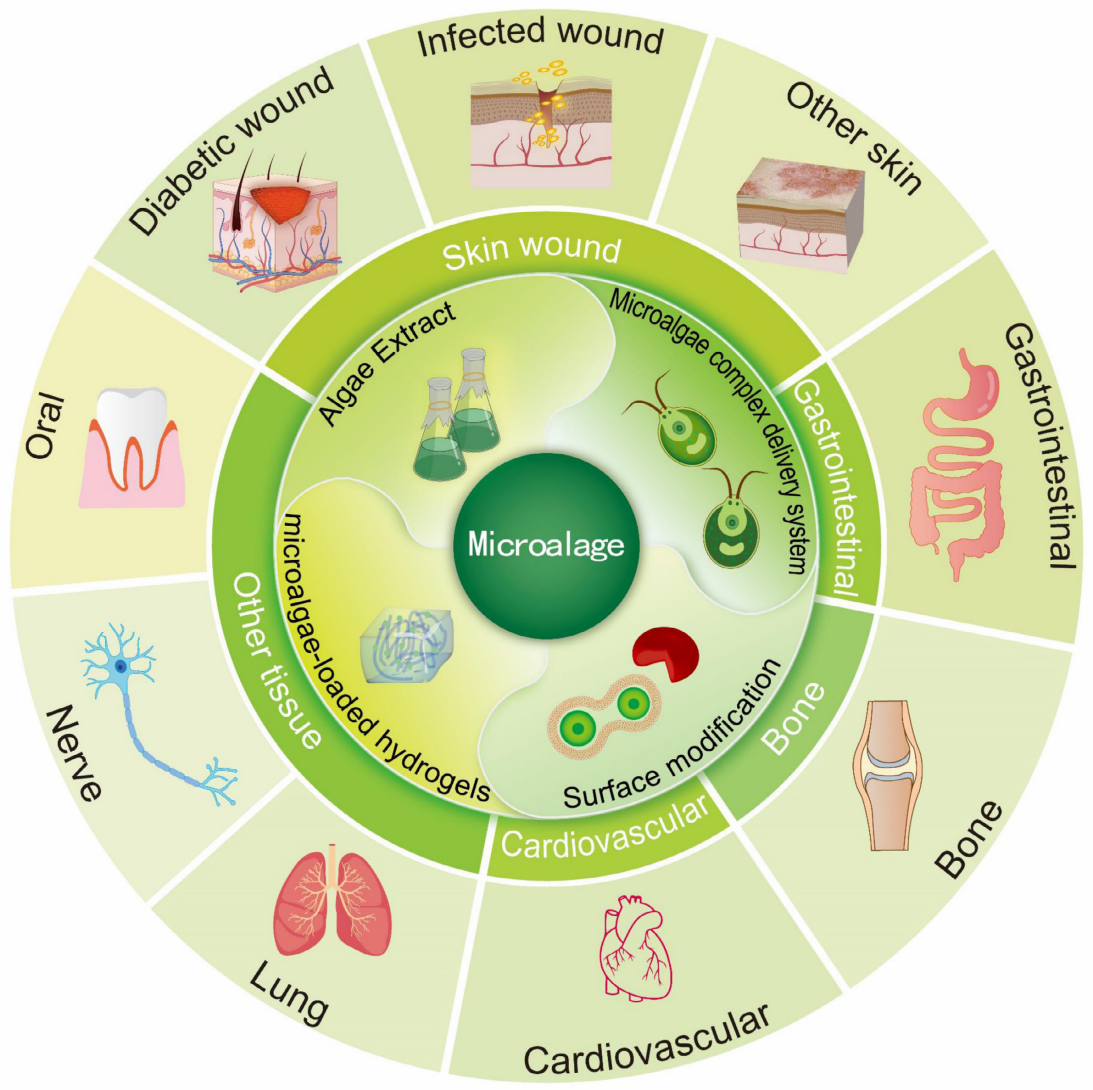
Microalgae in the biomedical field are commonly processed through methods such as bioactive substance extraction, the creation of microalgae-based composite drug delivery systems, surface modification, and living microalgae-loaded hydrogels. These processed forms are utilized to promote regeneration and repair across a range of tissues, including skin, gastrointestinal, bone, cardiovascular, lung, nerve, and oral tissues. (Created with Adobe lilustrator.com).

At present, a comprehensive review summarizing the applications of processed microalgae in the field of tissue repair is lacking. To address this knowledge gap, this review summarizes common processing methods used to process microalgae and discusses the advantages and disadvantages of different types of processed microalgae in tissue repair. In addition, recent research progress on microalgae and future research avenues are described, and the potential applications of processed microalgae in tissue repair are highlighted (Fig. 1).

## Microalgae classification and major medical applications

Microalgae vary in terms of species and morphological characteristics. Approximately 800,000 species of microalgae are found worldwide 12. However, only a few microalgae are used in biomedical applications. These microalgae can be classified as eukaryotic (mainly including *Chlorella*, *C. reinhardtii*, and diatoms) or prokaryotic (cyanobacteria, mainly *Spirulina*) on the basis of the presence or absence of chloroplasts, respectively 12.

### Chlorella

*Chlorella,* a spherical unicellular green alga, has a strong cell wall and is rich in intracellular components such as lipids, proteins, polysaccharides, and chlorophylls. It possesses antimicrobial, antioxidant, hydrogen-producing, and oxygen-producing properties 13,14. *Chlorellin*, derived from *Chlorella* extracts, has antimicrobial activity and can be used as an alternative to antibiotics in certain cases, preventing the development of drug resistance 15. *Chlorella* has high antioxidant levels and can promote tissue regeneration by scavenging reactive oxygen species (ROS) 16. In addition, it can convert solar energy to biohydrogen under anaerobic conditions, and this biohydrogen can be used as an antioxidant to reduce ROS levels, thereby alleviating oxidative stress and inflammation 17. Moreover, *Chlorella* has the ability to produce photosynthetic oxygen. *Chlorella* can act as an efficient oxygen producer for treating hypoxia-related diseases. In one study, the amount of oxygen produced by an autotrophic light-activated green oxygenation system composed of calcium alginate-coated *Chlorella pyrenoidosa* was three times greater than that produced by inorganic oxygen production materials. Consequently,* Chlorella* can promote tissue repair by improving the hypoxic tissue microenvironment and serve as a promising therapeutic agent for hypoxia-related diseases 18. Despite its benefits, the application of *Chlorella* in tissue repair faces certain challenges. The cell wall of *Chlorella* is notably robust, impeding efficient enzymatic breakdown in the human digestive tract and thus restricting nutrient absorption 19. Additionally, the extraction methods for active substances from *Chlorella vulgaris* are diverse, resulting in substantial variability in the potency of the resulting products 20. To fully leverage the therapeutic potential of *Chlorella* in tissue repair, additional research and technological progress are crucial.

### Chlamydomonas reinhardtii

*Chlamydomonas reinhardtii* (*C. reinhardtii*), a eukaryotic unicellular green microalga, is mostly spherical or ovate. Because its cell wall is negatively charged, it can be loaded with positively charged drugs or materials through electrostatic adsorption 21. The anterior end of *C. reinhardtii* has two equal-length flagella that can oscillate to swim directionally at a speed of 100 μm/s 22,23. This active movement helps promote drug diffusion and penetration in the human body, thus improving drug delivery efficiency. These attributes make *C. reinhardtii* a promising candidate for drug delivery systems. Additionally, the complete sequencing of the C. reinhardtii triad of genomes—nuclear, chloroplast, and mitochondrial—has been achieved. Through genetic engineering, a microalga gene recombinant expression platform can be constructed, facilitating the tailored design of its functional capabilities 24-26. For example, Jarquín-Cordero et al. developed a novel *C. reinhardtii* strain via a transgenic approach to produce the growth factor hVEGF-165, which promotes angiogenesis during wound healing 27. Although the gene editing technology of *C. reinhardtii* is relatively mature, its application still faces challenges. First, poor and inconsistent expression of nuclear transgenes remains an obstacle for both fundamental and applied research endeavors 28. Second, genetic manipulation has many disadvantages, such as strict codon usage, low transgene expression and gene silencing, and variable gene expression due to position effects 29.

### Diatoms

Diatoms, tiny unicellular microalgae with lengths ranging from a few micrometres to tens of micrometres, have a porous cell wall composed of silica. This porous structure allows the encapsulation of drugs, making diatoms promising vehicles for drug delivery 30. Coating the surface of diatoms with polydopamine (PDA) can prevent their degradation and removal in vivo and improve their viability 31. In addition, a novel nanomaterial formed by combining diatoms and graphene oxide has been shown to release chemotherapeutic drugs under acidic conditions for a localized effect, which can regulate the rate of drug release on the basis of the pH of the intestine, suggesting a novel strategy for the treatment of intestinal diseases 32. When diatoms die, their porous shells (i.e., silica) sink to the bottom of the water body. The accumulation of these shells eventually leads to the formation of diatomaceous earth, also referred to as biosilica 33,34. Owing to its three-dimensional porous and hollow structure and silanol groups on its surface, diatomaceous earth can concentrate coagulation factors and promote coagulation through endogenous pathways, thereby accelerating hemostasis 34,35. Compared with synthetic silica, diatomaceous earth has a larger microscopic size and is superhydrophilic and superhematotropic. Therefore, it can be used as a novel hemostatic material 36. While natural diatoms offer numerous advantages, the microscopic size of biosilica somewhat limits its route of administration. In areas with thin blood vessels and restricted blood flow, such as the vitreous cavity of the eye, biosilica tends to form a buildup. In addition, although intravenous biosilica is excreted by the kidneys, silica particles can still be detected in the liver, lungs, and glomeruli 37.

### Spirulina platensis

*Spirulina platensis* (SP), a typical spiral-shaped blue-green alga (200-500 μm in size), possesses anti-inflammatory and antioxidative properties 38. Excessive accumulation of ROS disrupts DNA structure, oxidizes proteins and lipids, delays tissue regeneration, and causes cell and tissue death 39-41. Therefore, the inhibition of ROS production or removal of excess ROS can attenuate inflammatory responses, reduce oxidative damage, and promote wound healing and tissue regeneration. C-Phycocyanin, which is extracted from SP, has demonstrated potent antioxidative, anti-inflammatory, and neuroprotective properties. It is a natural antioxidant that neutralizes ROS and reduces ROS-induced cell damage 42. Additionally, C-phycocyanin inhibits inflammatory responses and alleviates tissue damage, making it a promising drug or functional food for treating inflammatory diseases, oxidative stress, and neurodegenerative diseases 43-46. In addition, the surface and morphological features of SPs are very favorable for drug delivery. First, the negatively charged surface of SPs can be loaded with positively charged small-molecule drugs through electrostatic adsorption 47; second, the presence of water channels and connecting pore structures in the cell membrane facilitate the smooth entry of small-molecule drugs into the membrane 47,48; third, the spiral structure is conducive to embedding into the villi of the small intestine, thereby becoming a drug carrier with a relatively high loading rate, which enhances the intestinal drug delivery efficiency and bioavailability 47,48; fourth, the intrinsic fluorescence of chlorophyll within *Spirulina* enables noninvasive in vivo tracking without the need for additional fluorescent markers^48^. To fully harness the potential of *Spirulina* as a pharmaceutical carrier, several pivotal technical challenges must be addressed, including the enhancement of targeting precision, ensuring the stability of the carrier, guaranteeing its safety, and achieving controlled degradation under physiological conditions.

## Methods for processing microalgae

The biological functions of natural microalgae may be limited by immune clearance and the ability of light to penetrate human tissues. Processing approaches can improve the delivery efficiency and targeting ability of microalgae, thus enhancing their efficacy in tissue repair (Table 1). Currently, microalgae extracts, microalgae-nanodrug composite delivery systems, surface modifications, and living microalgae-loaded hydrogels are commonly used in biomedical applications. The combined use of different processing methods can further promote the multifunctionality of microalgae, providing a valuable reference for the future design and development of processed microalgae.

### Microalgae extracts

Microalgae extracts contain many bioactive substances, such as lipids, carbohydrates, and proteins 49. First, microalgae are rich in polyunsaturated fatty acids, such as eicosapentaenoic acid (EPA) and docosahexaenoic acid (DHA), which are necessary for nerve cells, and essential fatty acids, such as linolenic acid and linoleic acid 50. In 1944, microalgae fatty acids (*chlorellin*) were first extracted and shown to inhibit the growth of gram-positive and gram-negative bacteria 15. Since then, the antimicrobial effects of microalgal fatty acids have gradually attracted widespread attention. Researchers have attempted to assess whether microalgal fatty acids can be used as alternatives to classical antibiotics or whether their synergism with antibiotics can enhance their antibacterial effects. Among the major compounds extracted and purified from three microalgae (*Isochrysis galbana*, *Scenedesmus* sp.* NT8c*, and *Chlorella* sp.* FN1*) by Alsenani et al., linoleic acid, oleic acid, DHA, and EPA have been shown to inhibit the growth of gram-positive bacteria 51. Furthermore, microalgal lipids can be used for drug delivery. On the basis of their class and molecular stacking parameters, conventional lipids can be classified as lamellar or nonlamellar 52. Lamellar lipids, which have a self-assembled bilayer structure with one or more vesicular morphologies, are characterized by their ease of design, modeling, and similarity to biological membrane structures 52. Compared with lamellar liposomes, nonlamellar structures, such as cuboidal structures, have more complex configurations, higher surface area-to-volume ratios, and enhanced cargo encapsulation and sustained delivery 53-55. Clemente et al. extracted F&M-M24 lipids from *Nannochloropsis oceanica* and self-assembled them into cubes and liposomes for loading and delivery of natural antioxidants such as *curcumin*, *α-tocopherol*, and *piperine* 55. Second, in microalgae, carbohydrates are usually present in the cytoplasm and chloroplasts as cellulose, monosaccharides, polysaccharides, and starch, such as glucose, galactose, mannose, and arabinose 56. Microalgal polysaccharides exhibit antibacterial, antiviral, and antioxidative activities 57. The antimicrobial mechanism of microalgal polysaccharides is very complex. In addition to inhibiting bacterial adhesion, disrupting biofilms, and increasing cell membrane permeability, polysaccharides can act on bacterial ribosomes, affecting protein biosynthesis, metabolism, and nutrient uptake 58,59. Compared with unmodified polysaccharides, polysaccharide derivatives obtained through chemical modification (such as sulfation, carboxymethylation, and acetylation) of natural polysaccharides possess superior antimicrobial properties 60. In antiviral terms, the sulfated polysaccharides *p-KG03* 61, *Naviculan* 62, and *Ca-SP* 63 extracted from microalgae have been shown to possess antiviral properties. These polysaccharides exert antiviral effects by interfering with the viral life cycle 64. However, these effects strongly rely on the specific characteristics of the polysaccharide, such as the degree and type of sulfation, molecular weight, monosaccharide composition, three-dimensional structure, and hydrogen bond formation 64,65. In terms of antioxidative effects, the *Spirulina* polysaccharide complex (SPC) scavenges superoxide by upregulating superoxide dismutase 2 (SOD2) in aging fibroblasts, thereby restoring mitochondrial function and promoting collagen production to rejuvenate fibroblasts 66. In addition, microalgae are rich in proteins and other nutrients, including pigments, vitamins, and minerals such as carotenoids, chlorophyll, phycocyanin, vitamin A, vitamin E, folic acid, iron, and calcium. These components are used to treat various diseases, including cancer, cardiac disease, and immunodeficiency syndromes 67. For example, microalgae-derived β-carotene, a precursor of vitamin A, acts as an antioxidant 68. Phycocyanin, a microalgal-derived pigment protein, is a potential antioxidant, fluorescent molecular probe, and photosensitizer 69,70.

### Microalgae‒nanodrug composite delivery systems

In physiological media, natural drugs exhibit poor solubility, low bioavailability, and short effective action times, significantly weakening their therapeutic effects 71. Microalgae‒nanodrug composite delivery systems constructed from living microalgae provide an innovative and effective platform for drug delivery. Active microalgae, one of the most promising carriers for drug delivery, have negatively charged cell walls. Therefore, they can noncovalently bind to positively charged small-molecule drugs through electrostatic adsorption 72. In particular, the diatom cell wall, which is composed of silica and other components, can carry drugs and exhibit some resistance to strongly acidic reagents. Therefore, diatoms are not easily disrupted or digested 73. The surface and morphological characteristics of microalgae are conducive to improving the bioavailability of drugs. For example, small-molecule drugs can penetrate the membrane of *Spirulina* through water channels and connect pores (14-16 nm) on its surface, increasing the drug loading capacity 74. The unique flagellar structure of *C. reinhardtii* can generate strong propulsive forces in simulated intestinal fluids with sustained motility, facilitating the widespread distribution and retention of drugs in the small intestine 75. In one study, *C. reinhardtii* (flagellated) and static microalgae (nonflagellated) were encapsulated in protective capsules with internal hydrophobic and external enteric coatings, respectively, and their distributions in the gastrointestinal tract were assessed via fluorescence labeling after 5 h of oral administration. Compared with static microalgae, *C. reinhardtii* is more widely distributed in intestinal tissues. These findings indicate that the flagellar motility of *C. reinhardtii* plays an important role in enhancing its interaction with the intestinal wall and its ability to stay in the intestine 75. In contrast to spherical *Chlorella vulgaris*, helical *Spirulina* can be captured between the villi of the small intestine, increasing the retention time and bioavailability of drugs in the small intestine 47. Owing to their abovementioned properties and natural phototropism, microalgae have emerged as an important platform for constructing microalga‒nanodrug composite delivery systems. For example, the cell wall of *C. reinhardtii* is composed of glycoproteins enriched with 4-hydroxyproline (4-HP) residues. Weibel et al. achieved the binding of 4-HP glycopeptide-modified polystyrene particles to the surface of microalgae through noncovalent interactions. The particles are guided to reach the target site by the intrinsic phototropism of the microalgae and release polystyrene beads through photochemical reactions 76. The surface of microalgae is rich in chemical groups that serve as binding sites. These surfaces are commonly adorn with natural functional groups, such as carboxyl (-COOH) and amine (-NH2) groups, which originate from proteins or sugar components. By harnessing these inherent characteristics, the construction of stable covalent bonds can be efficiently achieved through methods such as amidation and click chemistry 77. For example, Zhang et al. utilized the amino group on the surface of microalgae to form an ester bond with N-hydroxysuccinimide (NHS) and reacted the drug with dibenzocyclooctyne (DBCO) via click chemistry to achieve covalent bonding between microalgae and the drug carrier 78. Overall, live algae are promising drug carriers that have attracted substantial attention in recent years owing to their unique structural properties, biocompatibility, self-propulsion, and phototropism.

### Surface modification

The widespread application of surface modification has provided novel opportunities in the biomedical field. Surface modification is crucial for the use of microalgae in the field of tissue regeneration and repair. Materials currently used to modify microalgae can be classified into cell membranes and magnetic nanomaterials. Cell membrane encapsulation is frequently used in studies. NPs are “camouflaged” into a cellular form by coating them with natural cell membranes through stirring, sonication, or mechanical extrusion. In this form, the proteins and polysaccharides present on the surface of the cell membrane can be used to protect the nanoparticles from immune clearance without affecting their inherent properties 79. Therefore, coating the surface of microalgae with a cell membrane can improve their circulation, targeting, and accumulation in vivo and effectively enhance their biocompatibility, thus improving their efficacy in tissue repair. On the basis of different requirements, cell membranes from different sources, such as erythrocytes, platelets, macrophages, and tumor cell membranes, perform different physiological functions. In particular, erythrocyte membranes can increase the blood circulation time and stability of microalgae 80. Platelet membranes can inhibit immune clearance 81 and actively target tissues or cells, such as tumor cells and damaged vascular tissues 82,83. Macrophage membranes can help evade immune clearance and actively target inflammatory and tumor tissues 84,85. Tumor cell membranes can specifically recognize and target corresponding tumors 86-88. Qiao et al. modified the surface of *Chlorella vulgaris* with a red blood cell membrane (RBCM) to design a novel biomaterial named RBCM-Algae via the stirring method. In vivo imaging revealed that compared with unmodified microalgae, RBCM-Algae effectively evades immune clearance and is efficiently delivered to tumor tissues owing to the presence of natural markers on the surface of RBCMs (e.g., CD47, sialic acid, and polysaccharides) 6. Additionally, ultrasonic treatment and mechanical extrusion are frequently employed techniques. Cheng et al. developed chlorella encapsulated within macrophage membranes for drug delivery applications. This was achieved by blending the cell membranes with chlorella, subjecting the mixture to ultrasonication for 2 minutes, and subsequently extruding it 40 times via a liposome extruder 84.

In addition, magnetic nanomaterials can also be used to modify microalgae. In recent years, many researchers have modified microalgae with magnetic nanoparticles by electrostatic adsorption or internalization and uptake of metal cations to achieve directional movement of microalgae under the action of an external magnetic field. For example, *Spirulina* has been modified with Fe_3_O_4_ via the dip-coating technique to achieve directional movement to the target site under the influence of an external magnetic field 89. Yasa et al. developed a biohybrid microswimmer by modifying *C. reinhardtii* with magnetic polystyrene particles (1 μm in diameter) through electrostatic adsorption for the targeted delivery of the particles under the influence of a magnetic field 22. Although magnetic nanoparticles can be attached to the surface of microalgae, this approach hinders cell movement and impedes drug loading on the surface of microalgae. To address this shortcoming, Santomauro et al. incubated microalgae with a medium containing terbium ions (Tb^3+^) by the internalization and uptake of metal cations, using functional groups on the cell wall to adsorb the ions. Small amounts of Tb^3+^ were translocated to the cytoplasm through internalization. Subsequently, magnetized microalgae containing Tb^3+^ were obtained by washing the cells with a dilute HCl solution and dissolving ions adsorbed on the cell surface 90. The inherent fluorescence properties of microalgae and the luminescent properties of Tb^3+^ can facilitate in vivo imaging. These findings suggest that surface modification of microalgae with cell membranes (e.g., erythrocyte, macrophage, and platelet membranes) or magnetic nanomaterials (e.g., Fe_3_O_4_, magnetic polystyrene particles, and terbium ions) can endow them with novel properties. Therefore, surface modification can help overcome the shortcomings of individual microalgae (e.g., immune clearance in vivo) and improve the targeting ability of processed microalgae in vivo for enhanced therapeutic effects.

### Living microalgae-loaded hydrogels

Hydrogel is a type of general-purpose soft material. Hydrogels can be divided into natural polymer hydrogels and synthetic polymer hydrogels according to the type of synthetic material. The gelators in natural polymer hydrogels are mainly natural polymers, such as starch, cellulose, alginate, and collagen. In contrast, gelators in synthetic polymer gels are mainly synthetic polymers, such as polyacrylic acid and polyethylene glycol 91. Currently, the majority of research endeavors have focused on the fabrication of living microalgae-loaded hydrogels through encapsulation within hydrogels and the utilization of 3D bioprinting techniques. These innovative approaches are predominantly leveraged in the realms of pharmaceutical delivery systems and tissue engineering. Gastrointestinal drug delivery systems constructed using living microalgae-loaded hydrogels can penetrate the gastrointestinal barrier. Ren et al. incubated insulin-loaded microalgae with sodium alginate and crosslinked them with calcium chloride to develop microalgae encapsulated with sodium alginate. This method preserves the inherent properties of microalgae and prolongs insulin retention in the intestine by preventing the rapid degradation of insulin in gastric acid 72. Hydrogels can mimic the extracellular matrix (ECM) and keep microalgae alive. Therefore, they can be used to load living microalgae or microalgae extracts for subsequent application in tissue repair. In addition, hydrogels can prevent infection in the early stage of skin injury, inhibit bacterial growth at the wound site, improve the local microenvironment, and support cell migration and differentiation, thereby promoting whole-layer skin repair. Chen et al. designed a microalgal gel patch capable of generating dissolved oxygen, which can be delivered to wounds to alleviate both acute and chronic tissue hypoxia, significantly facilitating wound healing 92. The rapid development of 3D bioprinting technology has revolutionized methods for processing microalgae, with 3D-printed hydrogels using activated microalgae as bioinks. Zhao et al. used 3D-printed filamentous protein hydrogels and *Platymonas* sp., a marine microalga, as bioinks. Experimental data have shown that hydrogels support microalgal proliferation for at least 4 weeks and maintain photosynthetic activity for more than 90 days 93. In situ 3D printing enables the direct application of living microalgae-loaded hydrogel scaffolds to damaged tissue sites. Wang et al. achieved in situ printing of microalgae-loaded hydrogel scaffolds on skin wounds via microfluidics in combination with 3D printing. This approach improved the bridging of hydrogels with the surrounding tissues and effectively accelerated the healing of chronic wounds 94. Living microalgae-loaded hydrogels can be developed into various structures according to specific requirements, such as microneedles, wound patches, and 3D scaffolds, offering the dual advantages of sustaining microalgae vitality and enabling functional modifications. The synergy between hydrogels and microalgae has significant therapeutic effects, including anti-inflammatory, antioxidative, and anti-infective effects and homeostasis regulation.

## Applications of processed microalgae in tissue repair

Processed microalgae play pivotal roles in enhancing drug targeting and therapeutic effectiveness and preserving the viability of microalgae. Many studies have demonstrated that microalgae can substantially improve therapeutic outcomes across various applications, including skin, gastrointestinal, bone, cardiovascular, lung, oral, and neural tissue treatments. In this section, we delve into the applications, design, therapeutic efficacy, and limitations of microalgae, aiming to provide insights into the current state and future directions of processed microalgae research in tissue repair (Table 2).

### Skin tissue

As the body's protective barrier, the skin protects against environmental damage. However, cutaneous wounds may lead to various local or systemic physiological and pathological changes, imposing a heavy burden on patients and society. Wound healing is a dynamic and continuous process involving multiple overlapping spatial and temporal phases, including hemostasis, inflammation, cell proliferation, and tissue reconstruction 95,96. During this process, different types of cells and biomolecules interact with each other to promote wound healing and restore the barrier function of the skin. Successful completion of both the spatial and temporal phases is necessary for achieving complete wound healing and restoring skin function 97,98.

#### Diabetic wound healing

The complex microenvironment of diabetic wounds, which are typically chronic, poses a serious challenge to healing. High levels of blood glucose lead to the accumulation of advanced glycosylation end products (AGEs), which exacerbate oxidative stress, suppress the phagocytic function of macrophages, and increase the secretion of inflammatory factors.

In addition, AGEs accumulate more neutrophils and macrophages in wounds 99. Excess macrophages and neutrophils increase the production of ROS and inflammatory factors, leading to chronic inflammation. Furthermore, inflammatory cells consume oxygen and nutrients, aggravating hypoxia and malnutrition 100,101. Oxygen plays indispensable roles in cell proliferation, neovascularization, collagen synthesis, and other processes involved in wound healing. Therefore, increasing the concentration of oxygen at the wound site can effectively accelerate wound healing 102. Localized rupture or constriction of blood vessels in the wound may impede the supply of oxygen, resulting in hypoxia, which is detrimental to wound healing. When the oxygen partial pressure decreases below the hypoxic threshold, the healing of chronic wounds is delayed 103. To address this challenge, localized or hyperbaric oxygen therapy is commonly used to treat chronic wounds in clinical settings. However, both of these traditional treatments have certain shortcomings. For example, the therapeutic effect of localized oxygen therapy is limited by the penetration of external gases into the skin, and only a trace amount of oxygen can enter the body through the skin and body fluids 104. In contrast to topical oxygen therapy, hyperbaric oxygen therapy involves the placement of patients in a high-pressure environment (for example, in a hyperbaric chamber), wherein they can inhale pure or highly concentrated oxygen to increase the partial pressure of oxygen and the content of blood and tissue oxygen, which promotes capillarization and wound healing. However, hyperbaric oxygen therapy needs to be performed in hospitals under the supervision of medical professionals, which greatly limits its application in the field of skin tissue repair 105,106. Therefore, ongoing research is focused on realizing prolonged oxygen delivery and improving the efficiency of oxygen delivery.

**Figure 2:**
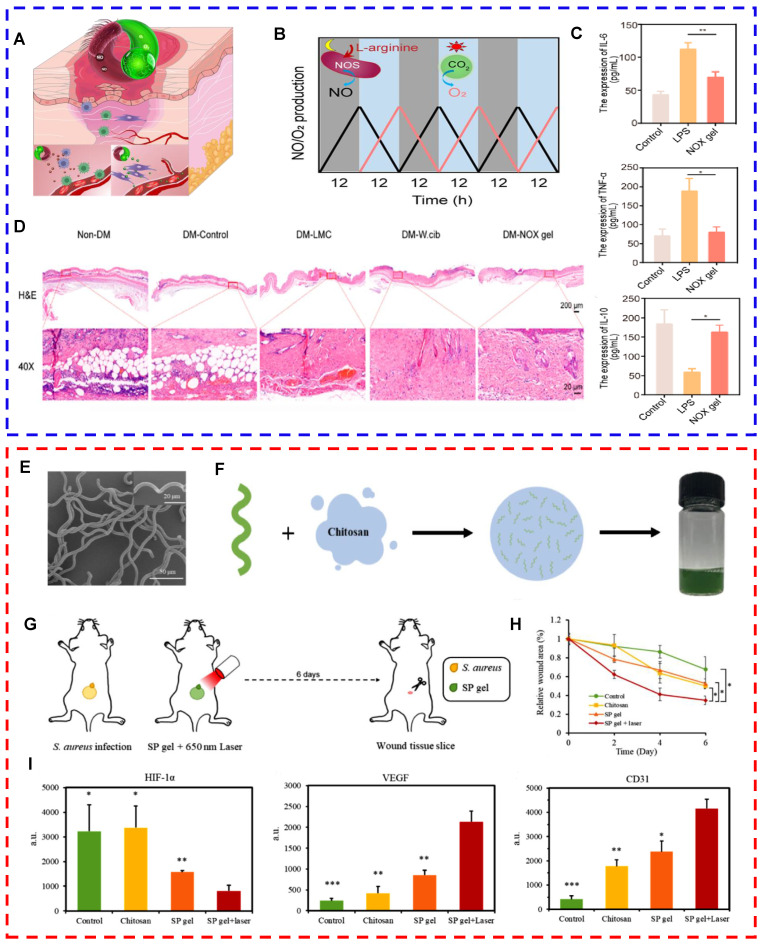
Oxygen-producing microalgal gel patches for repairing chronic diabetic wounds (reproduced with permission 108. Copyright 2023, American Chemical Society). (A) Mechanism of action of the NOX gel patch for the treatment of chronic diabetic wounds. (B) Functional regulation of NO (gray area) and O_2_ (blue area) production in lipid membrane-coated *Chlorella vulgaris* and* Weissella*. (C) Expression of IL-6, TNF-α, and IL-10 in different groups of LPS-induced RAW264.7 cells. (D) Representative images of diabetic wounds treated with or without NOX gel. (E-I) Microalgae-loaded hydrogels promoted the healing of infected wounds by improving the hypoxic microenvironment and exerting antimicrobial effects (reproduced with permission 120. Copyright 2020, WILEY-VCH Verlag GmbH & Co. KGaA, Weinheim). (E) SEM image of the SP gel. (F) Schematic illustration of the synthesis of the SP gel. (G) Schematic illustration of the in vivo experiments. (H) Measurement of the wound area at each time point and calculation of the relative wound area using the initial wound area on day 0. (I) Quantification of the results of immunohistochemistry (HIF-1α, VEGF, and CD31) using Image-Pro Plus to characterize the mechanisms through which the SP gel + laser group promoted wound healing.

Microalgae have gradually attracted attention as natural oxygenic organisms 107. Chen et al. developed a wound dressing containing living *Synechococcus elongatus PCC7942* and used it to heal chronic wounds in patients with diabetes. By leveraging the oxygen-producing photosynthetic capabilities of the microalgae, they created a localized moist environment with elevated oxygen levels, facilitating the delivery of dissolved oxygen to the wound. The penetration efficiency of this method was nearly 100 times greater than that of traditional localized oxygen therapy. The wound dressing effectively promoted oxygenation at the wound site and stimulated aerobic metabolism and angiogenesis in hypoxic tissues 92. Furthermore, Chen et al. applied a microalgal gel patch to skin graft wounds with poor microvessel formation and evaluated the effects of the patch on a mouse model of autologous skin grafts. The results revealed that mice treated with microalgal gel patches presented significantly increased microvessel density, intact epithelial structures, abundant granulation tissue, a large amount of well-aligned collagen, and a strong resemblance to the healthy epidermis of intact skin 92. Subsequently, Chen et al. developed a NOX hydrogel by integrating *Weissella* and *Chlorella*. They innovatively used nitric oxide synthase present in *Weissella* to catalyze the reaction of L-arginine with molecular oxygen to produce NO. Moreover, microalgae can act as an oxygen donor. The hydrogel unidirectionally delivered NO and O2 on the basis of circadian rhythms to alleviate chronic inflammation and hypoxia. The findings demonstrated that the hydrogel significantly reduced the expression of proinflammatory cytokines and improved neovascularization and tissue regeneration in a diabetic wound healing model and a diabetic flap transplantation model (Figs. 2A-D) 108. Expanding upon the foundational work of prior studies, our research team has also further explored the therapeutic potential of microalgae in the context of diabetic wound healing. We developed a novel hydrogel, denoted CHPS, which is a polyacrylamide‒sodium alginate hydrogel loaded with *Chlorella*. Upon exposure to 680 nm near-infrared irradiation, the sustained release of dissolved oxygen has been shown to markedly increase cell proliferation, migration, and angiogenesis 109.

As facultative anaerobic organisms, microalgae can convert solar energy to biohydrogen under anoxic conditions 110. Hydrogen acts as an antioxidant, selectively reducing the levels of hydroxyl radicals and peroxynitrite, promoting antioxidant enzyme expression, and decreasing inflammatory factor levels^111^,^112^. Traditional treatments such as hydrogen gas, hydrogen-rich water (HRW), and hydrogen-rich saline (HRS) have limited therapeutic efficacy owing to the short effective reaction time, explosive nature of hydrogen, and permeability of hydrogen to damaged tissues 17,113. However, as novel biomedical materials, microalgal hydrogel patches enable continuous generation and transdermal delivery of hydrogen to improve therapeutic efficacy. This approach can scavenge ROS, alleviate chronic inflammation at the wound site, and promote wound healing in diabetic wounds 17. Chen et al. proposed a symbiotic algal-bacterial wound dressing containing living *Chlorella vulgaris* and *Bacillus licheniformis*. The dressing functions through continuous consumption of oxygen in calcium alginate hydrogel beads by *Bacillus licheniformis* via the respiratory function of *Bacillus licheniformis*. Under hypoxic conditions, *Chlorella vulgaris* performs photosynthesis for 60 hours to produce hydrogen, thereby scavenging ROS and reducing -OH and ONOO- levels. The reduction in ROS levels promoted the polarization of macrophages to the M2 reparative phenotype and attenuated the inflammatory response. In vivo experiments revealed that on the third day after treatment initiation, the wound dressing promoted cell proliferation and led to approximately 50% healing of diabetic wounds 17. Processing microalgae for programmed treatment can improve the microenvironment of diabetic wounds. Kang et al. encapsulated live *Hematococcus* (HEA) in a conventional GelMA gel 114. During the experiment, high-intensity laser irradiation (658 nm, 0.5 W/cm^2^) was initially used to eliminate bacteria rapidly via the photothermal conversion effect. The light intensity (658 nm, 0.1 W/cm^2^) was subsequently reduced to allow the microalgae to continuously generate oxygen to ameliorate tissue hypoxia and promote vascular regeneration. Continuous light exposure promoted the accumulation of astaxanthin (AST) in HEA cells, effectively removing excess ROS. In addition, HEA cells further regulate macrophage polarization by secreting AST-rich vesicles 114. This programmed therapeutic strategy has a strong ability to regulate the wound microenvironment.

Overall, microalgae can promote the healing of chronic or diabetic wounds by producing oxygen or hydrogen through photosynthesis, exerting anti-inflammatory and antioxidative effects, and alleviating hypoxia. Wound dressings prepared using living microalgae-loaded hydrogels offer the following advantages: (1) the use of hydrogels to cover wounds creates a moist environment and provides a temporary barrier against external infections, and (2) this approach maintains the survival of microalgae and allows for the sustained delivery of dissolved oxygen to wounds in a moist environment to improve oxygenation. Patients with chronic diabetic wounds should be treated with a combination of hydrogels and microalgae‒nanodrug composite delivery systems to alleviate infection and inflammation. In addition, the photosynthetic activity of microalgae can increase oxygenation at the wound site, synergistically promoting wound healing.

#### Infected wounds

Wound infections pose a significant challenge in modern medicine, with millions of deaths attributed to untreated infections each year 115. The formation of infectious wounds is a complex process involving many factors, including the invasion of microorganisms, the host immune response, the activation of the inflammatory response, and possible complications 116. Antibiotics remain the mainstay of treatment for infections in clinical practice. However, poor targeting ability and nonselectivity to the focal area limit the efficiency of drug delivery. Achieving the required therapeutic dose often requires multiple administrations. Moreover, misuse of antibiotics tends to induce the formation of drug-resistant bacteria, resulting in less effective or ineffective treatment 117.

Processed microalgae offer various strategies for treating infected wounds, including the following: (1) Microalgae extracts exert antimicrobial effects. For example,* chlorellin*, which is extracted from *Chlorella*, can inhibit the growth of both gram-positive and gram-negative bacteria 15. Therefore, *chlorellin* can be used as an alternative to antibiotics to a certain extent, preventing the development of drug resistance. (2) Microalgae-nanomedicine composite drug delivery systems can be used to treat infected wounds. Shchelik et al. functionalized *C. reinhardtii* through azide cycloaddition using N-hydroxysuccinimide. In particular, they covalently attached vancomycin on the surface of the microalga via a cleavable o-nitrobenzyl 118. However, this antibiotic system does not function in the conjugated state. However, irradiation with 365 nm light disrupted the covalent bonds, resulting in the targeted release of the drug for the treatment of skin or soft tissue infections 118. (3) Microalgae rich in chlorophyll can generate ROS and function as photosensitizers for photodynamic therapy (PDT) under laser irradiation 119. Li et al. successfully prepared a hydrogel with photosynthetic oxygen production and antimicrobial activity via a one-step synthesis method. They utilized living *Spirulina* as the core material, encapsulating it within biocompatible carboxymethyl chitosan. The photodynamic bactericidal effects of *Spirulina* and the continuous release of dissolved oxygen improved the hypoxic microenvironment of the wound, alleviating infection and promoting healing (Figs. 2E-I) 120. (4) Magnetic nanocarrier-modified microalgae can be used for photothermal therapy (PTT) under laser irradiation. To date, no studies have used magnetic nanocarrier-modified microalgae to treat infected wounds. However, in terms of antimicrobial properties, researchers have used magnetite nanoparticles to modify the surface of *Spirulina*. Researchers have subsequently coated the surface of *Spirulina* with polydopamine to develop magnetic microalgal composites with improved performance. Under photoacoustic image guidance, these modified *Spirulina* materials possess magnetic properties and exhibit excellent photothermal activity. After 6 minutes of 808 nm laser irradiation, the microalgal composites can warm to >50 °C and exert antimicrobial effects 121.

Taken together, these findings indicate that microalgae possess excellent potential in the treatment of infected wounds. Microalgae extracts have antimicrobial properties. Microalgae rich in chlorophyll can generate ROS upon laser irradiation, enabling PDT for antimicrobial treatment. Additionally, microalgae can be loaded with drugs and magnetic nanoparticles, leveraging their magnetic properties for targeted movement and activation and achieving drug release or PTT.

#### Repair of other types of skin injuries

Microalgae have shown promising therapeutic effects against acute wounds caused by surgery and various inflammatory skin diseases (e.g., atopic dermatitis, psoriasis, and vitiligo). Lim et al. reported that the microalgae extract KSF0041 had oxygen radical-scavenging activity. KSF0041 effectively protected HaCaT cells against oxidative stress-induced damage 122. Furthermore, KSF0041 significantly alleviated clinical symptoms of psoriasis, including desquamation, redness, skin moisture loss, and weight loss, and inhibited inflammatory cytokine upregulation in imiquimod-induced psoriasis in mice 122. Liu et al. reported that spirochete protein (SPCP) promoted the repair of wounds caused by total dermal excision in C57BL/6 mice by upregulating the Akt, ERK, and TGF-β1 signaling pathways 123. With respect to surgical wounds, Centeno-Cerdas et al. pioneered the integration of genetically processed *C. reinhardtii* into surgical sutures, creating photosynthetic sutures. These innovative sutures enable the continuous and stable release of oxygen and recombinant human growth factors locally at the wound site, effectively promoting wound healing 124. This attempt not only led to the development of a new generation of bioactive sutures with increased regenerative capacity but also provided valuable insights into the development of microalgae-based biomaterials.

With respect to skin tissue engineering scaffolds, κ-carrageenan, a naturally sulfated algal polysaccharide derived from microalgae, has been found to be highly similar to the natural glucosaminoglycans (GAGs) present in the ECM of the skin 125. Singh et al. coated electrospun nanofibers with ECM-mimicking κ-carrageenan to construct skin tissue engineering scaffolds. The experimental data revealed that the scaffolds significantly promoted fibroblast adhesion, growth, survival, and proliferation and upregulated the expression of genes related to cell adhesion and cytoskeletal matrix formation. These findings indicated the formation of a favorable environment for fibroblast growth on the scaffolds 125.

As mentioned earlier, the use of microalgae to accelerate skin tissue repair is innovative, safe, and efficient. However, processed microalgae have many limitations in the field of skin tissue repair. At present, relevant research is at an early stage. In addition, the application of microalgae is limited mostly to the backs of experimental animals, which have relatively little movement. Wounds at these sites are significantly different from tension wounds at the neck, knee, elbow, or other joints. Resolution of these limitations is crucial for the clinical translation of microalgae.

### Gastrointestinal tissue

Oral administration is often the preferred route for the clinical treatment of gastrointestinal disorders. Microalgae offer the advantages of convenience and safety as oral medications. Fields et al. investigated the effects of consuming freeze-dried *C. reinhardtii* on gastrointestinal health in mice and humans. Experimental data revealed that mice with acute colitis fed PBS lost an average of 9% of their body weight, whereas those fed freeze-dried *C. reinhardtii* lost only 7% of their body weight after 12 days of treatment 126. These findings suggest that dietary supplementation with *C. reinhardtii* can mitigate colitis-associated weight loss. Furthermore, the addition of *C. reinhardtii* to the diet of patients with frequent gastrointestinal distress significantly reduced symptoms, facilitated regular bowel movements, and improved stool quality. In addition, when the gut microflora of these patients was examined, the consumption of *C. reinhardtii* was not found to have significant effects on the composition of gut microbes 126. Microalgae can also be used to treat heavy metal-induced tissue damage in the gastrointestinal tract. They can remove heavy metal ions from the body through electrostatic adsorption and chelation. Liu et al. engineered an innovative hydrogel, designated BBR-CV@ALG, for the remediation of lead intoxication. This composite material integrated *Chlorella vulgaris* (CV), berberine (BBR), and a composite of carboxymethyl chitosan and sodium alginate. The surface groups of *Chlorella vulgaris* can bind to lead ions, thereby exhibiting superior detoxification capabilities. Moreover, BBR mitigated the inflammatory responses induced by lead exposure through the neutralization of ROS. Moreover, the hydrogel formulation extended the gastrointestinal residence time of the microalgae, offering a novel in vivo detoxification approach for managing acute, subacute, and chronic lead toxicity 127.

**Figure 3:**
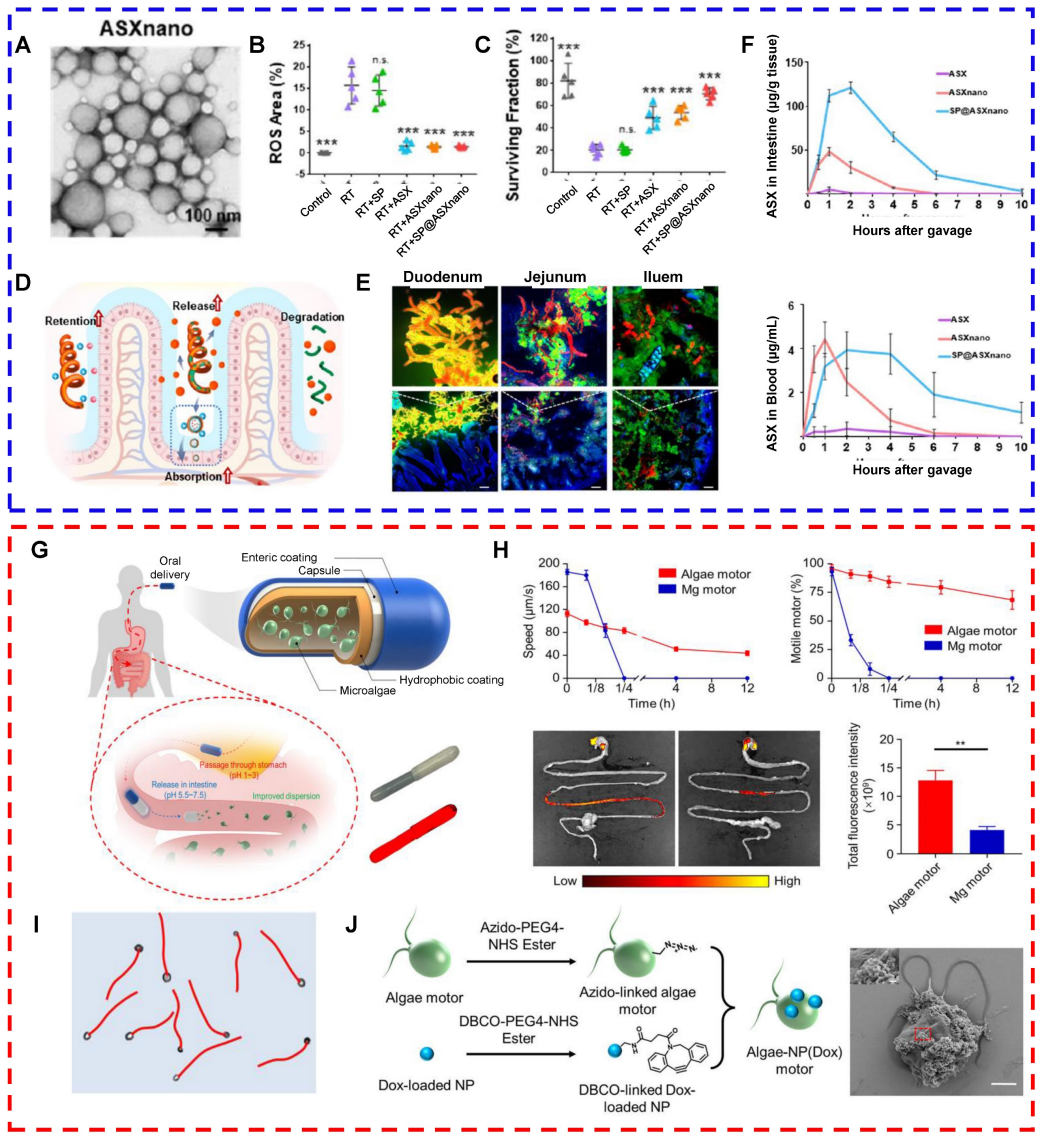
(A-F) Oral microalgae-nanointegrated system against radiation damage (reproduced with permission 132. Copyright 2023, American Chemical Society). (A) TEM images of ASXnano. (B) Fluorescence area measured on the basis of fluorescence (green) images of ROS in IEC-6 cells after 6-Gy X-ray irradiation (RT) of different materials for 6 hours. (C) Quantification of surviving colonies of IEC-6 cells after different treatments. (D) Schematic diagram of SP@ASXnano administration. (E) Fluorescence images of small intestinal sites 4 hours after the administration of SP@ASXnano via gavage. (F) Pharmacokinetic distribution of ASX in mouse intestinal tissues and blood after the administration of ASX, ASXnano, or SP@ASXnano via gavage. (G-J) Live algal nanorobots based on *C. reinhardtii* for gastrointestinal drug delivery (reproduced with permission 75. Copyright 2022, Author). (G) Schematic representation of algae motors loaded inside a protective capsule containing an internal hydrophobic coating and an external enteric coating for oral delivery; bright field and fluorescence images of the capsule containing the algal motor are shown. (H) Comparison of the distributions of algal and magnesium motors in the gastrointestinal tract. (I) Representative trajectories of the autonomous movement of algal motors in simulated intestinal fluid. (J) Schematic illustration and SEM images of the fabrication process of algal motors loaded with doxorubicin.

Moreover, a drug-carrying system based on living microalgae has been developed and used before and after radiotherapy to protect normal intestinal tissues. Radiotherapy, a conventional treatment for tumors, is administered to more than 70% of cancer patients 128. In addition to killing tumor cells, radiotherapy exerts toxic effects on normal tissues either systemically or locally, causing acute or chronic radiation syndrome and organ damage 129. For example, radiation therapy for tumors in the abdominal or pelvic cavity, such as pancreatic, prostate, and colorectal cancers, often damages the small intestine because of its high sensitivity to radiation and large size. Damage to the small intestine may lead to gastrointestinal dysfunction or death 130. Thus, minimizing radiotherapy-induced damage to the small intestine is essential. Drugs such as amphotericin have been approved to protect normal tissues from radiation. However, when these drugs are administered orally, they are readily affected by gastric acid and are rapidly metabolized, resulting in limited protective effects, a short half-life, and poor stability. Moreover, the potential side effects of these drugs limit their widespread application in clinical settings 131. Therefore, compared with traditional oral drug delivery systems, drug delivery systems constructed from living microalgae can overcome the limitations of traditional oral drug delivery, such as poor solubility, low bioavailability, and short half-lives of drugs 132. Zhang et al. constructed an oral microalga-nanointegrated system (SP@ASXNano) using *Spirulina platensis* (SP) as a drug delivery carrier and loaded astaxanthin nanoparticles (ASXNano). This system extended the median survival of mice with acute radiation enteritis to 29 days and ameliorated radiation-induced intestinal damage (Figs. 3A-F) 132. Moreover, compared with that of spherical algae, the spiral structure of Spirulina makes it better suited as a drug carrier. In one study, *SPs* loaded with curcumin were used to treat two intestinal diseases, namely, colitis and colon cancer. Researchers compared the retention times of *Chlorella vulgaris*- and *Spirulina*-based drug carriers in the intestine. A study revealed that *Spirulina*-based carriers were more likely to be captured by intestinal villi, prolonging the drug retention time and enhancing the absorption efficiency 47. Furthermore, microalgae can be loaded with drugs that are used to protect normal tissues from radiation, such as amifostine (AMF). Zhang et al. used a dehydration‒rehydration synthesis strategy to develop SP-based delivery vehicles for amifostine (SP@AMF). In vivo fluorescence imaging and SEM images of the gastrointestinal tract revealed that the SP@AMF carrier penetrated gastric tissues in an acidic environment and was more uniformly and widely distributed in the intestine. This system is capable of exerting a long-lasting effect, resulting in a comprehensive protective effect on the entire small intestine 130.

In contrast to *Spirulina*-based drug delivery systems, *C. reinhardtii*-based drug delivery systems offer certain advantages because of the unique flagellar structure of microalgae. This structure endows *C. reinhardtii* with the ability to swim rapidly and persistently in the intestinal fluid. It enhances the retention and absorption of drugs in the small intestine and improves the bioavailability of orally administered drugs. Therefore, *C. reinhardtii*-based drug delivery systems can be used for the diagnosis or treatment of gastrointestinal diseases. Zhang et al. developed nanorobots based on live *C. reinhardtii* with long-lasting self-propulsion capability. These nanorobots are capable of stable and continuous movement in the intestinal fluid at normal body temperature for more than 12 hours. The longer lifetime and more efficient motility of the algal motors than the metal micromotor facilitated the distribution and retention of drugs in the gastrointestinal tract (Figs. 3G-J) 75. Future studies should explore additional functionalities of algal motors to broaden their applications in gastrointestinal drug delivery. For example, photosensitizers such as chlorophyll in microalgae can enable visualization of the movement of microalgae as they function in the gut. In addition, the conjugation of magnetic particles to the surface of microalgae can more precisely direct the microalgal motors to the target site under the influence of an external magnetic field, enabling targeted delivery to specific regions in the gastrointestinal tract. These approaches may improve the precision and targeting ability of algal motor systems and enhance their efficacy in gastrointestinal drug delivery.

### Bone tissue

**Figure 4:**
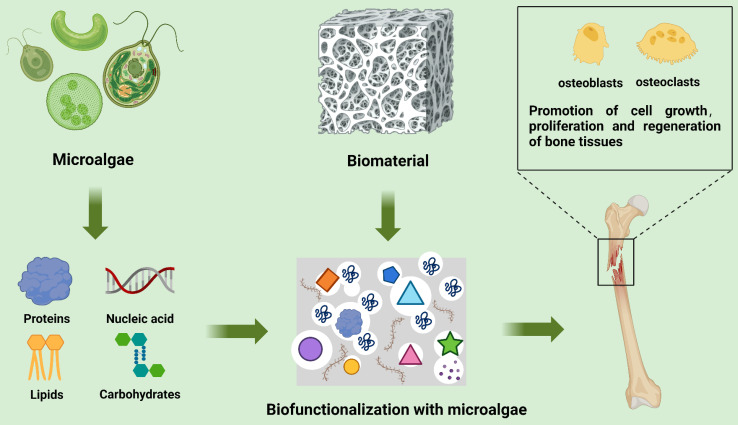
Schematic diagram of the combination of microalgae extracts (lipids, proteins, nucleic acids, and carbohydrates) and biomaterials for bone tissue repair 12 (created with BioRender.com).

To maintain the structural integrity of the skeleton, bones should be constantly remodeled. Osteoclasts remove old bone and are replaced by osteoblasts that synthesize new bone. Disruption of the balance between bone formation and bone resorption leads to disturbances in bone metabolism, which in turn triggers the development of several bone diseases, such as osteolysis, osteoporosis, osteoarthritis, and rheumatoid arthritis 133,134. Compared with individual microalgae, microalgae extracts exert more significant therapeutic effects against bone diseases. Astaxanthin, a component derived from microalgae, has been shown to have significant efficacy in improving the microstructure and thickness of bones 135. *Hematococcus pluvialis*, a natural antioxidant, is a rich source of astaxanthin. El-Baz et al. administered *Hematococcus pluvialis* (BHP, 450 mg/kg), the polar fraction (PHP, 30 mg/kg, nonastaxanthin component), and* Hematococcus pluvialis* extract (CHP, 30 mg/kg, enriched with astaxanthin) to osteoporotic rats orally for 14 days. Microcomputed tomography, serum biochemical tests, and other methods were subsequently used to compare the effects of the three treatments. The results showed that oral administration of BHP and PHP partially increased tibial bone mineral density and serum phosphorus levels while partially decreasing serum calcium, bone alkaline phosphatase, interleukin 6, osteoprotegerin (OPG), and nuclear factor-κβ ligand (RANKL) levels. Oral administration of CHP almost completely restored the abovementioned parameters to normal values, resulting in more significant therapeutic effects and confirming that astaxanthin inhibits osteoclast-mediated bone resorption 136. The ability of astaxanthin to increase the activity of osteoblasts has been demonstrated in an experimental model of periodontitis 137. For bone tissue repair, an ideal biomaterial should have excellent porosity, mechanical properties, drug-carrying efficiency, biocompatibility, biodegradability, antimicrobial activity, antioxidant activity, anti-inflammatory activity, and bone repair-promoting properties 138. Natural green microalgae can not only be extracted as bioactive substances but also be used as binders and pore-forming agents for the development of scaffolds for bone repair (Fig. 4) 12. Barua et al. constructed three new types of interconnected porous bone scaffolds via solvent casting. These scaffolds were composed of green microalgae and hydroxyapatite (HA) at ratios of 1:2, 2:1, and 1:1 (w/w). Comparative analysis revealed that scaffolds with equal microalgae-to-HA weight ratios (i.e., 1:1) had higher compressive and mechanical strengths, which met the requirements for cell growth and support 139. The silica shell of diatoms is a new type of biomaterial that holds substantial promise in bone repair. Owing to their complex structure, silica shells can be used as drug carriers with sustained drug release properties. Cicco et al. reported that sodium alendronate (NaALE)-loaded diatom silica shells not only improved the drug delivery rate but also prevented the side effects caused by prolonged systemic administration of drugs 140. Furthermore, the porous structure of diatom silica shells facilitates the adhesion and differentiation of osteoblasts. Amoda et al. sintered diatomaceous earth to construct a 3D cell growth platform for MC3T3-E1 preosteoblasts. The material exhibited excellent biocompatibility, with the cells beginning to attach to it by the second day. Von-Kossa staining revealed the presence of mineral deposits in osteoblasts after 21 days. The material was autoclaved and reused without leading to any adverse effects on subsequent cell culture 141. López-Álvarez et al. compared the effects of Si-HA coatings prepared from two silica sources (diatomaceous earth and quartz) and HA on a human osteoblast-like cell line (SaOS-2). An assessment of dsDNA and alkaline phosphatase (ALP) activity confirmed that Si-HA coatings prepared with diatoms were superior to those prepared with quartz in promoting the activity and proliferation rate of osteoblasts 142. The medicinal properties of microalgae, drug delivery efficiency, ability to stimulate bone tissue regeneration, and surface modification of scaffolding materials are considered key indicators of the efficacy of a biomaterial in bone repair 12. The development and design of different types of processed microalgae provide novel strategies for bone repair.

### Cardiovascular system

**Figure 5:**
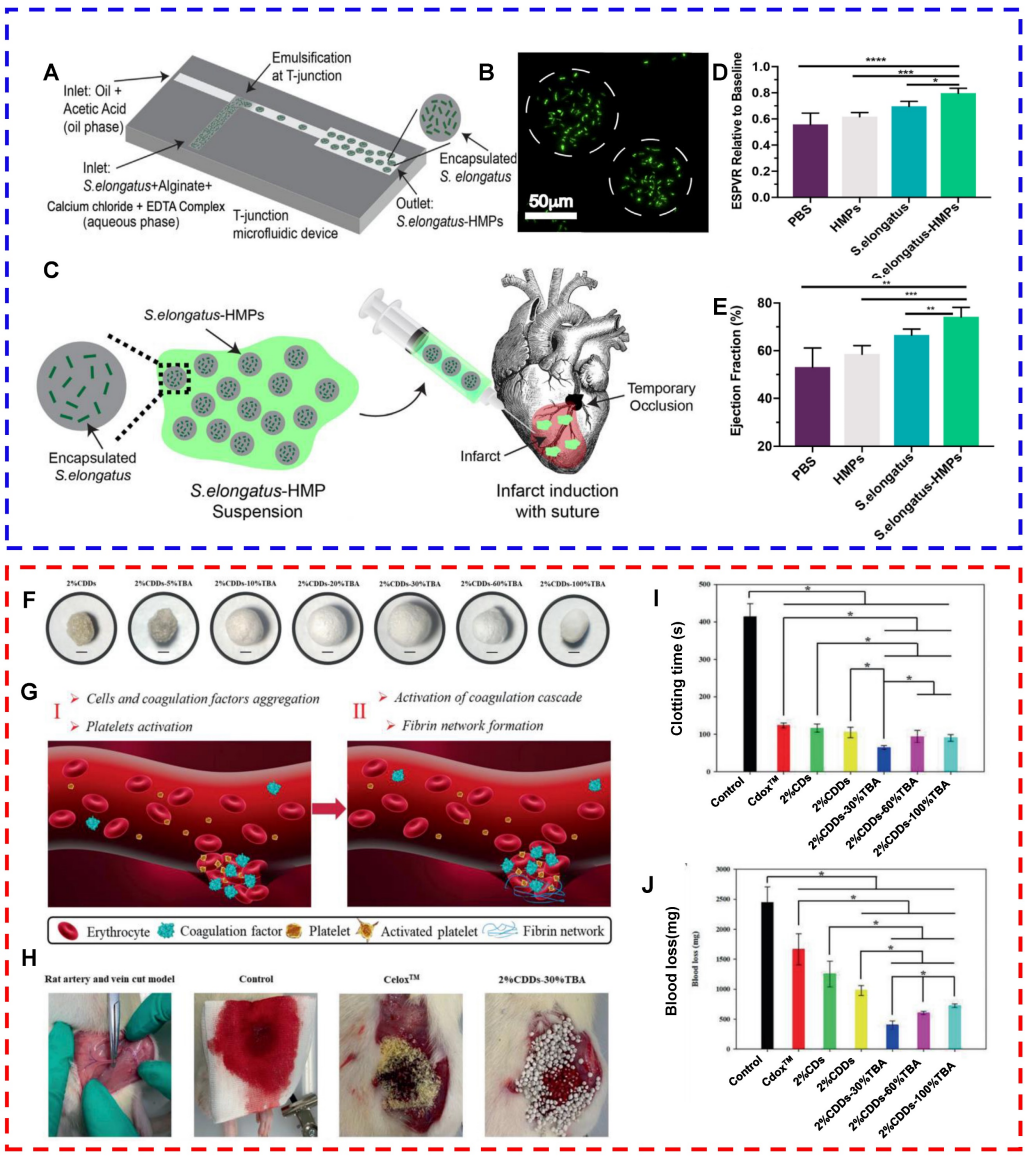
(A-E) *S. elongatus*-containing hydrogel particles (*S. elongatus*-HMPs) for the repair of ischemic myocardium (reproduced with permission 147. Copyright 2023, The Society for Biotechnology, Japan. All rights reserved). (A) Schematic diagram of the microfluidic device used to fabricate *S. elongatus*-HMPs. (B) Microscopy image of *S. elongatus* encapsulated in HMPs. (C) *S. elongatus*-HMPs delivered to the ischemic myocardial tissue stopped bleeding by temporarily blocking the left anterior descending (LAD) artery. (D) ESPVR values obtained by comparing the systolic pressure‒volume relationship (ESPVR) values of each animal with the ESPVR values at baseline after 45 minutes of treatment. (E) Effects of *S. elongatus*-HMPs on the ejection fraction 4 weeks after ischemia-reperfusion. (F-J) Chitosan/Diatom-Biosilica aerogels (CDDs-TBA) for rapid hemostasis (reproduced with permission 35. Copyright 2020, Wiley-VCH GmbH). (F) Representative images of 2% CDDs, 2% CDDs-5% TBA, 2% CDDs-10% TBA, 2% CDDs-20% TBA, 2% CDDs-30% TBA, 2% CDDs- 60% TBA, and 2% CDDs-100% TBA after freeze-drying. (G) Formation of clots at vascular wounds after the application of CDDs-TBA. (H) Representative images of the hemostatic effects of CDDs-TBA. (I) Clotting time. (J) Bleeding volume in a rat model of femoral arteriovenous dissection.

Cardiovascular diseases (CVDs) involve lesions of the myocardium and vascular tissue, including myocardial infarction (MI), atherosclerosis, and heart failure. A deficiency in oxygen supply is a critical etiological factor for numerous cardiovascular conditions, with particular emphasis on the scenario of profound myocardial hypoxia. In such cases, the ischemic regions are at risk of sustaining irreversible damage, potentially leading to necrosis 143-145. Owing to their photosynthetic oxygen production activity, microalgae represent revolutionary and promising therapeutic agents for hypoxia-related CVDs. Microalgae can convert carbon dioxide to oxygen during myocardial ischemia, thereby improving the oxygen deficit in the microenvironment, rescuing ischemic cardiomyocytes, and maintaining their survival 146. To increase the survival of microalgae in the human body, Stapleton et al. designed alginate-based hydrogel particles (*S. elongatus*-HMPs) containing* Synechococcus elongatus* (*S. elongatus*, a type of cyanobacterium) 147. In vitro experiments revealed that the cyanobacteria in HMPs survived longer than those suspended in a phosphate-buffered solution. In addition,* S. elongatus*-HMPs significantly alleviated cardiomyocyte hypoxia and reduced cardiomyocyte apoptosis. In vivo experiments revealed that *S. elongatus*-HMPs suppressed apoptosis and improved left ventricular function in mice with ischemia‒reperfusion injury (Figs. 5A-E) 147. EPA and DHA found in microalgae can reduce the morbidity and mortality rates of CVD. Microalgae-derived peptides with antioxidative and anti-inflammatory properties may alleviate hypertension and regulate dyslipidemia by inhibiting angiotensin-converting enzyme (ACE) and endothelial nitric oxide synthase 148,149. Chen et al. assessed the protective effects of a peptide, *I. zhanjiangensis* (PIZ, FEIHCC), derived from the marine microalga *Isochrysis zhanjiangensis,* which is a golden-brown flagellate species isolated from Nansan Island, Zhanjiang, Guangdong Province, China. This evaluation focused on its potential to mitigate endothelial damage in human umbilical vein endothelial cells (HUVECs) 150. The results showed that PIZ (IC50 = 61.38 μM) antagonized ACE in a noncompetitive binding manner and inhibited angiotensin II (Ang II)-induced secretion and expression of vascular factors by blocking the NF-κB, Nrf2, MAPK, and Akt signaling pathways 150. When surgery or trauma leads to severe damage and rupture of arterial or venous vessels or when specific bleeding wounds develop (e.g., irregular wounds, deep luminal wounds, and multiple tiny oozing wounds), traditional hemostatic strategies, such as tourniquets and hemostatic agents, may not be able to control the bleeding effectively. Diatomaceous earth has silanol groups on its surface and a multistage porous structure that can rapidly absorb plasma and bind to blood components (i.e., red blood cells and platelets), thereby concentrating coagulation factors and facilitating coagulation. Therefore, diatomaceous earth is a novel hemostatic biomaterial 35,151. Using dopamine as a cross-linking agent, Li et al. developed chitosan/diatom-bBiosilica aerogels (CDDs-TBA) via the "alkaline precipitation-tertiary butanol replacement-freeze-drying method". The results revealed that CDD-TBA had a large specific surface area (74.441 m2 g^-1^) and a high water absorption rate (316.83 ± 2.04%), with a clotting time of <80 s in vitro. The therapeutic effects of CDDs-TBA were subsequently examined in a rat tail-breaking model and a femoral arteriovenous dissection model. Compared with commercial products, CDD-TBA led to significant improvements in the clotting time and bleeding volume metrics (Figs. 5F-G) 35. As mentioned earlier, processed microalgae-based strategies for cardiovascular repair can alleviate hypoxia, control coagulation, and mitigate the impact of CVD risk factors. In addition, these strategies can improve the quality of life of patients with CVD.

### Other tissues

**Figure 6:**
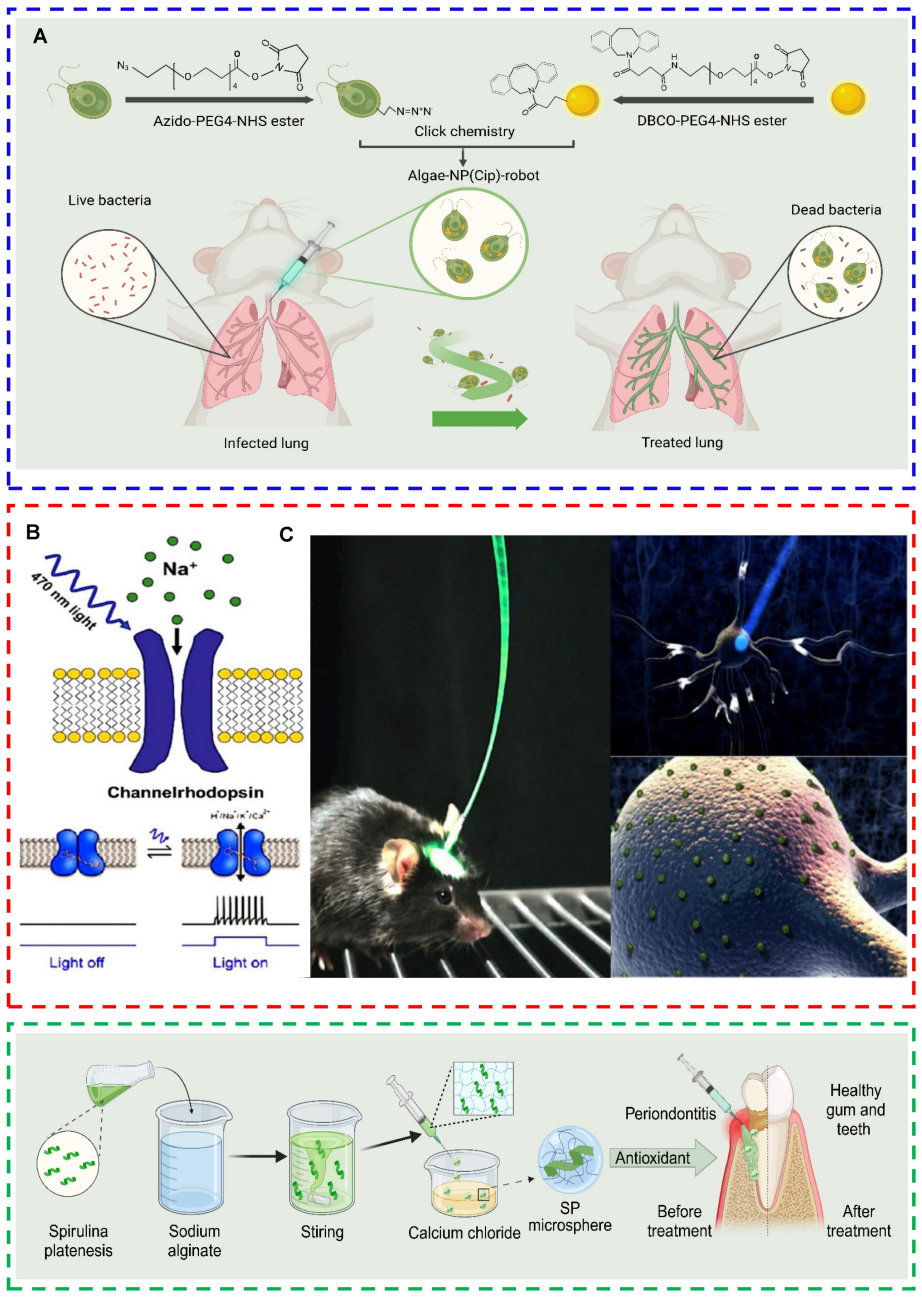
Microalgae are processed for the repair of lung, nerve, and oral tissues. (A) Schematic diagram of a microalga‒nanodrug composite delivery system that can actively deliver drugs to the lungs 78 (created with BioRender.com). (B) Schematic diagram of the photosensitive channel protein rhodopsin. (C) Light transmission to the rodent brain via optical fibers, neuronal activation after light stimulation, and expression of ChR2 on the membranes of neurons (reproduced with permission 157. Copyright 2013, Elsevier B.V.). (D) Schematic illustration of the synthesis process for microalgae-based hydrogel microspheres and their application in chronic periodontitis therapy 160 (created with BioRender.com).

The applications of processed microalgae extend to several tissues other than those mentioned above, such as lung, nerve, and oral tissues. For the treatment of lung injury, microalgae‒nanodrug composite delivery systems can efficiently encapsulate drugs and deliver them to lung tissues. These systems can improve drug bioavailability and overcome the limitations imposed by the unique physiological barriers and defense mechanisms of the lungs, such as mucociliary clearance in the upper respiratory tract and phagocytosis by macrophages in the alveoli 78. Zhang et al. functionalized the surface of *C. reinhardtii* via azido N-hydroxysuccinimide (NHS) ester, followed by conjugation with dibenzocyclooctyne (DBCO)-modified neutrophil membrane/polymer NPs via click chemistry. They successfully constructed a microalgae-nanoparticle microrobot (algae-NP-robot) for drug delivery to the lungs. Owing to the motility of the flagella of *C. reinhardtii*, algae-NP-robots presented high drug delivery efficiency and improved the therapeutic efficacy of the drug (Fig. 6A) 78. On the basis of the autofluorescence characteristics of algal chloroplasts, fluorescence imaging of isolated lungs was performed at different time points to observe the distribution of algae-NP-robots. The results revealed that the algae-NP-robots penetrated the whole lung tissue within 1 h and were retained for at least 24 h, with an even distribution throughout the lung tissue 78. Microalgae extracts are the predominant processed microalgae used in the neurological field. Most microalgal extracts (e.g., omega-3, C-phycocyanin, and fucoxanthin) intervene with or repair neuronal cell damage by alleviating oxidative stress 152, inhibiting neuroinflammation 153,154 and promoting neuronal cell growth 155. Among microalgae extracts, the photosensitive ion channel protein ChR2 extracted from *C. reinhardtii* has attracted substantial attention owing to its ability to manipulate the activity of cation channels in neuronal cells. ChR2, analogous to a circuit switch, manipulates the initiation and inhibition of various physiological activities in the neurons and muscle cells of living organisms 156. Boyden et al. used lentiviral vectors to transfect laboratory-cultured neuronal cells with ChR2 and irradiated the transfected cells with blue light. The ChR2 protein captures light, allowing extracellular Ca^2+^, Na^+^, and other cations to enter nerve cells and cause excitation, thus enabling the manipulation of nerve cell activity with millisecond-level temporal precision 156. On the basis of these findings, the effects of ChR2 on different mouse tissues were examined. The results showed that ChR2 controlled cardiac muscle contraction and promoted the recovery of respiration after SCI by activating neural circuits. Furthermore, ChR2 was introduced into retinal ganglion cells or cochlear cells to improve visual or auditory perception, respectively (Figs. 6B-C) 157. Microalgae have shown strong potential in the repair of oral tissues 158. Yuce et al. evaluated the effects of astaxanthin, a component derived from microalgae, on alveolar bone in a rat model of ligation-induced periodontitis. They measured changes in alveolar bone levels and performed immunohistochemical staining. The results revealed that astaxanthin promoted osteoblast-mediated bone formation, suppressed osteoclast-mediated bone resorption, and significantly reduced alveolar bone loss in rats 137. Owing to the moist and dynamic environment of the oral cavity (e.g., temperature changes, salivation, swallowing, and chewing) and the presence of various bacteria 159, orally administered microalgae extracts have a short local retention time and inefficient drug delivery capacity. To address these issues, Kaipa et al. engineered a spirulina-enriched hydrogel microsphere specifically designed for targeted treatment of chronic periodontitis. These microspheres facilitate the direct delivery of spirulina to affected oral regions, leveraging its antioxidant properties to significantly ameliorate this condition 160 (Fig. 6D). Taken together, the processing of microalgae may endow them with desirable properties for the repair of lung, nerve, and oral tissues. However, further research is warranted to assess the application potential of different processing techniques.

## Conclusion

In this study, the applications of processed microalgae in tissue repair were systematically summarized. The extraction of organic matter from microalgae can improve the bioavailability of their active components and enhance therapeutic efficacy. Microalgae-based composite drug delivery systems can improve the delivery rate and bioavailability of drugs. Surface modification of microalgae with cell membranes or nanomaterials can help them evade immune clearance and enhance their targeting ability in vivo. Living microalgae-loaded hydrogels can maintain the activity of microalgae for improved therapeutic effects. In addition, microalgae have good metal adsorption capacity and can be used as biocarriers to adsorb and remove heavy metals such as lead and mercury from the body, thus alleviating heavy metal poisoning. Owing to the presence of fluorescent chlorophyll pigments, microalgae can be used for fluorescence imaging in vivo and can be combined with PDT. Moreover, the distinct characteristics of various tissues and organs necessitate a tailored approach. Consequently, microalgae offer a diverse array of future research avenues, specifically for the treatment of particular diseases or tissues. For example, in the treatment of skin wounds, the focus should be on increasing local oxygen concentrations to increase the efficiency of oxygen production. Given the limited light conditions within internal organs, the integration of upconversion luminescent materials or wireless luminescent devices is essential for addressing hypoxic diseases in vivo. In the context of gastrointestinal and pulmonary diseases, attention must be given to the targeting and drug-loading capabilities of microalgae as drug carriers, as well as their self-luminescent properties, to increase the precision of disease diagnosis and treatment. Concurrently, in the realm of bone tissue engineering, the development of microalgae-based scaffolds represents a promising research direction.

In addition, the synergistic application of microalgae with biomaterials could encompass future advancements such as functionalization, customization, and the application of genetic engineering techniques. First, functionalization is an important direction for the development of processed microalgae, especially for drug delivery. Endowing microalgae with specific functions by processing methods can improve drug delivery efficiency and treatment efficacy, thus promoting more comprehensive and effective tissue repair. Second, in the current era of precision medicine and digitalization, the demand for processed microalgae is increasing. Processed microalgae that are "tailor-made" according to the specific conditions of each patient and the diverse requirements of tissue repair can facilitate individualized treatment. For example, introducing photosynthetically living microalgae into hydrogel scaffolds or dressings via 3D printing can enable individualized treatment. 3D printing can be used to combine drug-loaded microalgae and hydrogels and customize them with various drug release properties, such as the ability to release drugs in response to external stimuli (e.g., temperature, pH, and light). This design allows processed microalgae to flexibly adjust the release of drugs in response to changes in the external environment for different therapeutic requirements. Third, genetic engineering of microalgae is another important direction for future research. Precise modification of microalgae through genetic engineering can enable them to produce more bioactive substances (e.g., proteins, lipids, carotenoids, and biohydrogen) or have specific functional expression, improving their efficacy in tissue repair.

Although the prospect of utilizing processed microalgae in tissue injury repair is encouraging, research into their efficacy in promoting healing also faces several challenges. First, studies investigating the applications of processed microalgae in tissue repair are insufficient. Efforts to improve the survival of microalgae in organisms and increase their ability to accumulate at target sites effectively are ongoing. Second, the majority of microalgae processed for tissue repair applications remain confined to the preclinical experimental stage. In addition, existing clinical studies on processed microalgae have focused mostly on microalgae extracts (Table 3), whereas clinical studies on other processed microalgae are relatively limited. To verify the application value of processed microalgae in clinical treatment, it is necessary to conduct clinical studies on more types of processed microalgae. In addition, the optimal therapeutic dose and expected therapeutic effects of processed microalgae and the duration of treatment warrant further investigation. Future research should focus on processed microalgae to maximize their potential as biomaterials. Processed microalgae may introduce novel avenues for tissue repair and regeneration and promote the continuous development of microalgae in the biomedical field.

## Figures and Tables

**Table 1:** Processing methods for microalgae

Engineering strategy	Classification	Processing options	Advantage	Disadvantage	Operational difficulties	Applicable scenarios	Refs.
Microalgae extract	Lipids extraction	Conventional organic solvents extraction	Simple operation, low cost	Long extraction time; high toxicity of some organic solvents (e.g. chloroform and methanol)	Extraction efficiency is often influenced by operating conditions such as temperature, pressure, microalgal state (concentration, dry/wet state, growth stage), and scale	Drug carriers; nutraceuticals and biomedical applications	161
		Ionic liquids extraction	Low toxicity, flexible synthesis, nonvolatile, thermally and chemically stable	High cost, only small-scale preparation			
		Supercritical fluid extraction (e.g. supercritical C02 extraction)	Nontoxic, nonhazardous, low cost, mild critical pressure, and low critical temperature	The low polarity of carbon dioxide makes it harder to penetrate the polar cell membranes and hard cell walls of microalgae.			
		Accelerated solvent extraction	Low solvent consumption, fast and efficient	requirements, high energy consumption			
		Pulsed electric fields	Simple operation	High energy consumption			
		Ultrasound-assisted extraction	Simple operation, energy-saving, and high efficiency	The intensity and time of ultrasound need to be controlled to avoid negative effects			
		Microwave-assisted extraction	Fast and efficient	The polarity of the solvent significantly affects the extraction efficiency and selectivity			
		Enzyme-assisted extraction	mild operating conditions, energy-saving.	High cost; it is necessary to optimize the conditions to get the highest extraction rate			
	Protein extraction	1. Ball milling method	Simple equipment, gentle and efficient	High energy consumption	Effective cleavage of cell walls, protein recovery, issues such as product purity, energy consumption, and nondestruction of active substances during the extraction process	Nutraceuticals and biomedical applications	162-168
		2. Ultrasound-assisted extraction techniques	Simple operation, energy-saving, high efficiency	Small-scale preparation, thermal effects affect protein quality			
		3. High-pressure homogenization	High efficiency	High energy consumption			
		4. Pulsed electric field treatment	Gentle, efficient, high protein purity	Low extraction rate, high energy consumption			
		5. Aqueous enzymatic method	Mild operating conditions, specialization	Long extraction time, high cost, and difficulty in selecting specific enzymes for each microalgae;			
		6. Ionic liquids extraction	Low volatility, good stability	High cost			
		7. Repeated freezing method	Simple operation, high stability of proteins	Long extraction times, small-scale preparation			
		8. Supercritical fluid extraction	Short extraction time, high efficiency	Low extraction rate, high cost, low extraction rate			
		9. Microfluidization	High efficiency	High cost, low extraction rate, high equipment requirements			
	Polysaccharides extraction	Hot-water extraction methods	Simple operation; low cost	Long extraction time, low extraction rate and purity	Extraction efficiency is often influenced by operating conditions such as extracting times, time, PH, etc.	Nutraceuticals and biomedical applications	169,170
		Alkali extraction methods	High extraction efficiency	High cost,			
		Ultrasound-assisted extraction techniques	Simple operation, energy-saving, and high efficiency	Easy to damage polysaccharide structure			
		Microwave-assisted extraction	Simple operation, energy-saving	Easy to damage polysaccharide structure			
		Repeated freezing method	Simple operation	Low-efficiency, small-scale preparation			
		Enzyme-assisted solvent extraction	Simple operation, high efficiency, mild operating conditions, protecting polysaccharide bioactivity	Unstable enzyme activity			
	Pigments extraction	The traditional methods: solid‒liquid extraction, liquid‒liquid extraction, and Soxhlet extraction; The innovative methods: Supercritical fluid extraction, Pressurized liquid extraction, Microwave-assisted extraction, Ultrasound-assisted extraction, and Enzyme assisted extraction, etc.	Innovative approach: less time-consuming, less solvent consumption, large amounts of purified extraction fractions	Traditional methods: lower efficiency, longer time, toxicity, higher solvent consumption	Organic solvents pose environmental and safety risks; enzyme-assisted extraction requires control of specificity and stability	Nutraceuticals and biomedical applications	171
Microalgae-based composite drug delivery systems	Covalent bonds	1. Amidation reactions 2. Click Chemistry	Improved stability of microalgae drug delivery systems	Irreversible; affects microalgal activity	Requiring specific reaction conditions, complex processing	Drug delivery	77,172
	Noncovalent Bonds	Electrostatic adsorption	Reversible, allowing dynamic and controlled assembly and disassembly of biological systems	Less stable than covalent bonds	Vulnerable to factors such as pH, temperature, and ionic strength, etc.	Drug delivery	77,173
Surface modification	Cell membrane encapsulation	1. Stirring method 2. Ultrasonication 3. Mechanical extrusion	Prolonged circulation of microalgae in vivo; targeting; preservation of cell membrane surface antigens	Immune cell membranes encapsulating microalgae may induce or exacerbate inflammation through interactions with the body's immune system.	Stirring method has low fusion efficiency; the fusion efficiency frequency of the ultrasonication method is limited by time and vibration amplitude; the Mechanical extrusion method is unsuitable for large-scale production.	Targeted therapy, drug delivery, in vivo fluorescence imaging	6,84,174
	Magnetic nanoparticles modification	1. Electrostatic adsorption 2. Internalization and uptake of metal cations	Easy to assemble; highly precise motion capability under remote external magnetic field control; noninvasive dual-modality imaging using autofluorescence and MRI signals	A higher concentration of magnetic nanoparticles inhibits microalgae growth; Loaded with magnetic nanoparticles; Inhibits the deposition of therapeutic agents on the microalgae's surface.	The efficiency of random interactions between microalgae and magnetic nanoparticles is suboptimal; The internalization of metal cations is constrained by factors such as their concentration, the duration of incubation, and the density of microalgal cells.	Targeted therapy, drug delivery, in vivo fluorescence imaging, Photothermal inhibition	22,90,175,176
Living microalgae-loaded hydrogels	Hydrogel-encapsulated	A multifunctional bioactive hydrogel system is formed by loading active microalgae into a hydrogel matrix.	Good biocompatible; providing an aqueous environment for embedded microalgae, maintaining microalgae activity	Restriction of cell growth	Precise control of reaction conditions such as pH, concentration, temperature, etc.	Drug delivery, wound dressings, applying to gastrointestinal injuries due to heavy metal poisoning	172,177,178
	3D bioprinting technology	Utilizing microalgae in conjunction with hydrogel substances (e.g., sodium alginate, carrageenan, etc.) to develop bioinks, allows for the 3D printing of biological materials with sophisticated three-dimensional configurations	Good biocompatible, providing a three-dimensional culture environment, maintaining microalgae activity, customizable shape	The printing process reduces the survival of microalgae; complex processing	Stabilization of bioinks; control of printing parameters; maintenance of microalgal activity	Drug delivery; tissue-engineered scaffolds	179,180

**Table 2:** Role of processed microalgae in the field of tissue regeneration and repair.

Processing strategy	Microalgal species	Processed microalgae	Cellular or animal mode	Role and mechanism	Refs.
Microalgae extract	Red alga	κ-carrageenan	NIH 3T3 cells	Bionic skin ECM; promoting the adhesion, growth, survival, and proliferation of fibroblasts and upregulating genes related to cell adhesion and cytoskeletal matrix formation	125
	*Hematococcus pluvialis*	Astaxanthin	Osteoporotic rats	Inhibition of osteoclast-mediated bone resorption	136
	*Isochrysis zhanjiangensis Microalgae*	Polypeptide	Human umbilical vein endothelial cell vascular injury model	PIZ antagonizes ACE in a noncompetitive binding manner and inhibits the Ang II-induced secretion and expression of vascular factors by blocking the NF-κB, Nrf2, MAPK, and Akt signaling pathways	150
	*Porphyridium* sp.	Polysaccharides	Human coronary artery endothelial cell inflammation model	Inhibition of NF-κB activation and TNF-α-induced oxidative stress in HCAECs	181
	*C. reinhardtii*	Channel rhodopsin-2	Retinal degeneration mouse model; cochlear cells	Modulation of cation channel activity in nerve cells, modulation of neuronal and muscle cell activity, and improvement of visual or auditory perception	^.^^157^,^182^
	*Spirulina*	*Spirulina* protein	Full-thickness skin excision wound mouse	Modulation of the Akt, ERK, and TGF-β1 signaling pathways promotes skin wound repair in mice	123
	*Spirulina*	*Spirulina* extract	Dextran-sulfate-sodium induced ulcerative colitis in rats	Hydroalcoholic extracts of *Spirulina* attenuate dextran sulfate sodium-induced inflammation	183
Microalgae-based composite drug delivery systems	*C. reinhardtii*	Microalgae--nanoparticle microbots	Acute pseudomonas aeruginosa pneumonia mouse	Improved efficiency of drug delivery to the lungs and uniform distribution throughout lung tissues	78
	*C. reinhardtii*	*C. reinhardtii* loaded with Adriamycin	Oral administration to mice	Promoting drug distribution and retention in the gastrointestinal tract by using the efficient and long-lasting motility of *C. reinhardtii* and the protective ability of oral capsules	75
	*C. reinhardtii*	Chitosan-heparin nano complex coated microalgae	Chronic diabetic mice wound	Oxygen production via photosynthesis and anti-inflammatory effects; capable of penetrating moderately dense blood clots to reach deep wounds	184
	Diatoms	Diatom silica shells loaded with sodium alendronate	J774 cells; Bone marrow stem cells; Human SaOS-2 osteoblastic cell line	Increased drug delivery rates	140
	*Spirulina*	Magnetic *spirulina* composites constructed from ferrite nanoparticle-modified *Spirulina*	Infection mouse model	Magnetic properties, photothermal properties, and antimicrobial effects	121
	*Spirulina*	*Spirulina platensis* loaded with astaxanthin nanoparticles	Rat small intestinal epithelial cells; acute radiation enteritis mouse	Extension of median survival of mice with acute radiation enteritis to 29 d and amelioration of radiation-induced intestinal damage	132
	*Spirulina*	SP-mediated delivery of amifostine	Rat small intestinal epithelial cells; early intestinal radiation injury mouse; delayed intestinal radiation injury mouse; orthotopic colorectal cancer irradiated mouse	The SP@AMF drug delivery system can pass through the acidic gastric environment and distribute more evenly and widely in the intestinal tract, exerting comprehensive protective effects on the entire small intestine	130
Surface modification	*C. reinhardtii*	Noncovalent bonding of magnetic polystyrene particles with *C. reinhardtii*	NIH 3T3 cells; HeLa cells; OVCAR-3 cells.	Realization of targeted therapy in the presence of an external magnetic field	22
	*C. reinhardtii*	*C. reinhardtii* loaded with terbium ions (Tb^3+^)	Human breast cancer cells; mouse fibroblasts	Realization of directional movement under the influence of a magnetic field and in vivo fluorescence imaging	90
Living microalgae-loaded hydrogels	*Chlorella vulgaris*	Algal gel patches prepared using *Chlorella vulgaris* and *Bacillus licheniformis*	Diabetic mouse wound	Sustained hydrogen production under anaerobic conditions; promotion of diabetic chronic wound healing in vivo by alleviating oxidative stress and inflammation	17
	*Chlorella vulgaris*	Coencapsulation of *Chlorella vulgaris* and *Weissella* in calcium alginate hydrogels	Diabetic mouse wound	Alleviation of chronic inflammation and hypoxia by alternating NO and O_2_ production, reduction in the expression of pro-inflammatory cytokines, and improvement in neovascularization and tissue regeneration	108
	*Chlorella vulgaris*	Polyacrylamide-sodium alginate hydrogel loaded with *Chlorella*	Diabetic mouse wound	The sustained release of dissolved oxygen, augmenting cell proliferation, migration, and angiogenesis	109
	*Chlorella vulgaris*	Microneedles constructed using *Chlorella* and antimicrobial polyionic liquid	Infected diabetic mouse wound	The continuous production of oxygen by *Chlorella* via photosynthesis and the bactericidal activity of PIL promote wound healing	185
	*Spirulina*	Natural polymer carboxymethyl chitosan-coated *spirulina*	Infected diabetic mouse wound	Oxygen production via photosynthesis and continuous production of dissolved oxygen; Chlorophyll in *Spirulina* produces ROS under light to exert an antimicrobial effect	120
	*Spirulina*	SP@Rh-gel hydrogels prepared using *Spirulina* and rhein	Chronic colitis mouse model	Improved drug bioavailability; inhibition of the NF-κB-caspase-1 signaling pathway reduces intestinal inflammation and maintains intestinal homeostasis; reduction in the release of pro-inflammatory cytokines and lipopolysaccharides and preventing their penetration through the blood‒brain barrier, thereby inhibiting neuroinflammation	186
	*Spirulina*	*Spirulina* hydrogel microspheres	Chronic periodontitis patients	Antioxidant	160
	*Spirulina*	Hydrogel containing 12% *Spirulina* and 20% chitosan	Diabetic rat tooth extraction wound	Downregulation of the pro-inflammatory factors IF-1β and TGF-α and upregulation of the anti-inflammatory factor IF-10 promote wound healing after tooth extraction	187
	*Chlorella pyrenoidosa*	Photosynthetic microalgae scaffolding material	Chronic diabetic murine wound	Oxygen production via photosynthesis, accelerated neovascularization, and collagen deposition; real-time matching and rapid repair of tissue defects of any shape and depth	94
	*S. elongatus*	Alginate hydrogel particles loaded with *S. elongatus*	A murine model of myocardial ischemia	Relief of cardiomyocyte hypoxia and reduction of cardiomyocyte apoptosis	147
	*S. elongatus PCC7942*	Microalgae hydrogel patch	Chronic diabetic murine wound	Oxygen production and delivery of dissolved oxygen to the wounds to promote angiogenesis	92
	HEA	GelMA hydrogel loaded with microalgae HEA	Infected diabetic mouse wound	Oxygen production via photosynthesis, ROS scavenging, antimicrobial effects, anti-inflammatory effects, and modulation of macrophage polarization for the overall rapid healing of diabetic wounds	114
	*Microalgae*	A carbomer gel containing microalgae, basic fibroblast growth factor, and covalent organic framework	Diabetic mouse wound	Oxygen production via photosynthesis, ROS scavenging, anti-inflammatory effects, and promoting angiogenesis	188
Microalgae extract; Living microalgae-loaded hydrogels	Diatoms	Chitosan-coated diatomaceous earth	Rat-tail amputation model	Improved biocompatibility, shortened clotting time, and reduced bleeding	189
Microalgae-nanodrug composite delivery system; Living microalgae-loaded hydrogels	*Chlorella vulgaris*	Preparation of composite hydrogels using *Chlorella vulgaris*, Berberine, and carboxymethyl chitosan-sodium alginate	Chronic lead poisoning mouse	Adsorption and removal of metal ions from the body; extended retention time of microalgae in the gastrointestinal tract by hydrogels	127

**Table 3:** Clinical trials related to the use of microalgae extracts in tissue repair. (Source: https://clinicaltrials.gov/)

Clinical trial code	Experimental phase	Condition or disease	Interventions/treatments	Trial purpose
NCT04851899	Not Applicable	Cognitive function	Participants were administered oral placebo or microalgae extract PhaeoSOL for 1 month	To assess whether PhaeoSOL composition affects the cognitive function of participants
NCT04832412	Not Applicable	Cognitive impairment and neuroprotection	Participants were orally administered maltodextrin as a placebo or the microalgae extract BrainPhyt for 24 weeks	To assess the effects of BrainPhyt on cognitive function (spatial working memory scores, attention and alertness, situational memory, and executive function), stress, mood, sleep quality, and biomarkers in healthy older adults
NCT05759910	Not Applicable	Age-associated memory impairment	Participants were orally administered maltodextrin capsules as a placebo or the microalgae extract Brainphyt capsules for 12 weeks	To assess the effects of the microalgae extract BrainPhyt on cognitive function in healthy older adults
NCT05267301	Phase 4	Heart health	Participants received the oral dietary supplement AlmegaPL, an EPA-rich microalgae extract, for 6 months	To assess the effectiveness of Almega PL in improving blood markers associated with heart health over the period of evaluation
NCT01437930	Not Applicable	Cardiovascular disease	Participants were orally administered a placebo or microalgae-derived n-3 PUFA	To assess the effects of dietary supplementation of n-3 PUFA on cardiovascular risk factors in patients with hypertriglyceridemia
NCT00728338	Not Applicable	Cardiovascular disease	Patients were orally administered olive oil as a placebo or microalgae-derived DHA	To evaluate the effects of dietary supplementation of DHA on cardiovascular risk factors in male patients with hyperlipidemia
NCT03625284	Not Applicable	Nonalcoholic fatty liver disease	Participants were orally administered capsules of edible oil as a placebo or microalgae-derived fucoxanthin capsules	To investigate the effects of microalgae-derived fucoidan on biochemical clinical markers related to liver health
NCT01742468	Not Applicable	Rheumatoid arthritis	Participants were orally administered sunflower oil as a placebo or microalgae-derived long-chain n-3 PUFA for 10 weeks	To assess the effects of microalgae-derived long-chain n-3 PUFA on disease activity, inflammatory markers, and cardiovascular risk factors in patients with rheumatoid arthritis
NCT03960164	Phase 1	Acute Wounds	The integration of *Chlamydomonas reinhardtii* microalgae within Evicel human fibrin sealant constructs a dermal regeneration photosynthetic matrix (DRPM)	To assess the effects of DRPM on the treatment of acute wounds

## Abbreviations

ACE: angiotensin-converting enzyme
AGEs: advanced glycosylation end products
ALP: alkaline phosphatase
algae-NP-robot: a microalgae-nanoparticle microrobot
AMF: amifostine
Ang II: angiotensin II
AST: astaxanthin
ASXNano: astaxanthin nanoparticles
BBR: berberine
BHP: *Hematococcus pluvialis* biomass
CDDs-TBA: Chitosan/Diatom-Biosilica aerogels
ChR2: Channel rhodopsin-2
CHP: *Hematococcus pluvialis* extract rich in Astaxanthin
CHPS: a polyacrylamide-sodium alginate hydrogel loaded with *Chlorella*
*C.reinhardtii*: *Chlamydomonas reinhardtii*
CV: *Chlorella vulgaris*
CVDs: Cardiovascular diseases
DBCO: dibenzocyclooctyne
DHA: docosahexaenoic acid
DRPM: dermal regeneration photosynthetic matrix
ECM: extracellular matrix
EPA: eicosapentaenoic acid
GAGs: glucosaminoglycans
HA: hydroxyapatite
HEA: *Hematococcus*
HRS: hydrogen-rich saline
HRW: hydrogen-rich water
HUVECs: human umbilical vein endothelial cells
MI: myocardial infarction
Na ALE: sodium alendronate
NHS: N-hydroxysuccinimide
OPG: osteoprotegerin
PDA: polydopamine
PDT: photodynamic therapy
PHP: The polar components of *Heamatococcus pluvialis*
PIZ: *I. zhanjiangensis*
PTT: photothermal therapy
RANKL: nuclear factor-κβ ligand
RBCM: red blood cell membrane
ROS: reactive oxygen species
*S. elongatus*: *Synechococcus elongatus*
*S. elongatus*-HMPs: *Synechococcus elongatus*-containing hydrogel particles
SOD2: superoxide dismutase 2
SP: *Spirulina platensis*
SPC: *Spirulina* polysaccharide complex
SPCP: spirochete protein
SP@AMF: Amifostine-loaded *Spirulina platensis*
SP@ASXNano: Astaxanthin nanoparticles-loaded *Spirulina platensis*
4-HP: 4-hydroxyproline
